# Community-minded, adolescent-driven: addressing behavioral health disparities through youth participatory research designs

**DOI:** 10.3389/fpubh.2026.1771899

**Published:** 2026-08-07

**Authors:** J. Maya Hernandez, Tessa Palafu, Summer Pascual, Zoe Primack, Gustavo Madrigal, Miki Tomita Okamoto, Hye Jung Kim Tano, Paloma Almanza, Coco Besson, Mizuko Ito, Anna C. Ciao, Ijeoma Opara, Kelsie H. Okamura

**Affiliations:** 1 Wellesley Centers for Women, Wellesley College, Wellesley, MA, United States; 2 Perelman School of Medicine, University of Pennsylvania, Philadelphia, PA, United States; 3 Independent Consultant, Kailua, HI, United States; 4 Department of Psychology, University of Oregon, Eugene, OR, United States; 5 Department of Psychology, University of California, Los Angeles, Los Angeles, CA, United States; 6 ListoAmerica Inc., Tustin, CA, United States; 7 Education Incubator, Honolulu, HI, United States; 8 Shuksan Middle School, Bellingham, WA, United States; 9 Department of Informatics, University of California, Irvine, Irvine, CA, United States; 10 Department of Psychology, Western Washington University, Bellingham, WA, United States; 11 School of Public Health, Yale University, New Haven, CT, United States; 12 Leonard Davis Institute of Health Economics, Philadelphia, PA, United States

**Keywords:** adolescence, behavioral health, community partnerships, health disparities, youth-centered, YPAR

## Abstract

Ongoing intergenerational trauma, racial injustice, technological advancements, and the COVID-19 pandemic contribute to increased mental health risks and a persistent unmet need for youth evidence-based behavioral health services. While research and intervention implementation continue to progress, it is rare to see efforts that intentionally include adolescent perspectives to bring evidence-based programming to communities. Youth-centered approaches such as Youth Participatory Action Research (YPAR) offer researchers and systems leaders an orientation to learn through the youth voices most impacted, building programs in developmentally informed ways while simultaneously promoting positive youth development. This paper showcases the participatory research impacts by presenting four unique community case studies that illustrate processes to dismantle behavioral health disparities in California, Washington, Hawaiʻi, and New Jersey. In each case, adolescents and the communities that serve them collaborate, co-research, and act as agents of change to address specific mental health-promoting topics. Drawing on our collective years of engaging directly with young people in applied research across diverse environments, this brief case study collection offers collaborative perspectives of participatory research relationship-building, sustainable and time-conscious programming, and team creation that promotes bidirectional knowledge transfer for shared decision-making. The authors explore overlapping contextual considerations influencing participatory trust, relationship hierarchies, collaborative decision-making around research and programming, and creating sustainable behavioral health promotion activities anchored in community. This paper also acknowledges the deteriorating funding landscape for behavioral health initiatives and offers guidance for financially sustainable program co-creation. Authored by researchers and community partners, these case studies provide actionable frameworks that are scalable and adaptable to new communities and implementation teams that address key adolescent developmental tasks and cultural considerations. Integrating youth perspectives directly into rigorous research processes represents an applied solution to pressing issues affecting education, communities, and the future of adolescent behavioral health.

## 1 Introduction

A significant surge in behavioral health concerns among developing adolescents has been recognized over the past decade, prompting US Surgeon Generals to declare a growing youth mental health crisis (1). Complex factors such as intergenerational societal and political events, natural disasters, racial injustice, technological advancements, and residual effects of the COVID-19 pandemic all contribute to increased risk for youth mental health disorders (1). The heightened risk coupled with unmet evidence-based youth behavioral health services needs further amplifies this public health priority (2). Progress in intervention research and implementation has done little to center adolescent perspectives, hindering the ability to address persistent health disparities—measurable differences in health outcomes between specific populations—and limiting adaptations of evidence-based programming to the unique needs of diverse contexts (2).

Adolescence, as defined as the period following the onset of puberty toward adult development, is a critical period for psychosocial development including behavioral changes that may influence long term trajectories of wellbeing (3). As adolescents (also referred to as “teens” or “youth” throughout this paper) explore identity, bodily changes, and online ecosystems, exposure to risks increase as priorities shift from family to peer relationships. Persistent health and healthcare access disparities impact this stage of development, despite calls for the field to meet youth where they are which includes digital spaces (2). Furthermore, communities (i.e., groups of people linked by social ties, shared common perspectives, and collective action in a specific geographical or online setting) face systemic oppression and obstacles related to healthcare access, creating unmet needs and opportunities to promote positive youth outcomes (4, 5). These disparities are even more striking for youth who live in racially, socioeconomically, and geographically diverse communities. Mistrust in research, social services, government, education, and private sectors may amplify gaps. These gaps are further hindered by deficits-based orientations to intervention and prevention research that limit our ability to highlight youth as experts of their lived experiences.

### 1.1 Youth participatory approach and ladders of participation

Adult experts often make assumptions about fundamental adolescent experiences, which rapidly evolve depending on their environment and sociopolitical systems. Participatory research puts youth in the lead by harnessing their strengths, including their direct perspectives and holds promise for impactful behavioral health systems change. Youth Participatory Action Research (YPAR) is a youth-adult partnered approach in which youth identify problems relevant to their lives, conduct research, and share their findings to change systems meant to serve them and their communities (6). This offers opportunities for researchers and systems leaders to collaborate with youth partners, historically those who are most impacted by health disparities, to translate research into opportunities for U. S youth health solution-building (6–8). YPAR also promotes positive youth development and agency through acknowledging youth as the underrepresented experts of their lived experiences (9, 10). However, a review of the YPAR literature revealed that most studies focused on academic or civic engagement outcomes, with limited to no studies focused on emotional and behavioral outcomes (11). Therefore, work to address behavioral health disparities remains vastly underrepresented in participatory research. By shifting power dynamics from researchers to youth, participatory approaches in research can function as an opportunity to decolonize and foster liberation from traditional hierarchies in behavioral health (12). It simultaneously provides a scaffold for adolescents to navigate critical developmental tasks such as psychological autonomy, a sense of purpose, and exploration of collective identities (13).

Programs designed to center youth as the main change agents serve as a support system for youth during this dynamic developmental period. However, YPAR approaches are not the only approach that positions youth as the experts (14, 15). Hart describes participatory practices through the ladder ranging from non-participation (e.g., tokenism, decoration, manipulation) to participation (e.g., consulted and informed, youth initiated and directed, shared decisions with adults). Sustainment becomes central to the research process by actively involving youth throughout and avoiding non-participation (16). Youth voice is elevated through power redistribution in teams with variation depending on the extent to which youth initiate, lead, or are consulted on projects.

### 1.2 How youth participation reduces behavioral health disparities

Youth participatory research can serve as a catalyst to reduce behavioral health disparities with youth either leading the process (6) or partnering with adults in co-design and evaluation practices (17, 18). A systems-level pathway can improve accessibility of programs meant to serve youth, while an individual-level pathway can strengthen behavioral health literacy, help-seeking, and normalizing behavioral health with youth and their networks (6, 7, 14).

At the systems level, youth can identify a behavioral health concern salient in their own communities, investigate through research, and translate their findings into culturally grounded solutions that are disseminated in systems that they are engaged in, such as schools, clinics, and community organizations (6, 7, 14). Because solutions are generated by and for youth who experience the disparity, resulting programs are more responsive to context and more accessible, which address structural and access-related dimensions of behavioral health disparities (7, 17). This process also deconstructs Eurocentric focus of typical research that often minimizes youths’ agency and identities tied to their surrounding social and cultural environments (12, 14). Furthermore, the experience of participating itself functions as behavioral health promotion. By reinforcing youth autonomy, confidence, and connection in research, participation builds behavioral health literacy, reduces stigma, and empowers youth to advocate for behavioral health support with their peers and families (6, 10, 13, 19). Youth become knowledgeable messengers with skills to narrow behavioral health disparities by becoming direct agents of change (YPAR) or building intentional youth-adult partnerships to ensure the youth voice is heard in solution-building processes (19, 20).

These pathways are driven by core adolescent developmental tasks such as psychological autonomy, purpose, and collective identity, which allows participatory approaches to remain adaptable across levels of participation while striving for long-term systemic change for diverse contexts, cultures, and behavioral health needs (6, 14, 17, 20). This paper presents four brief youth participatory case studies by scholars and their community partnerships. Each illustrates various processes, outcomes, and limitations as adolescents and their communities serve as collaborators, co-researchers, and agents of behavioral health.

## 2 Context: case studies overview

**Figure 1:**
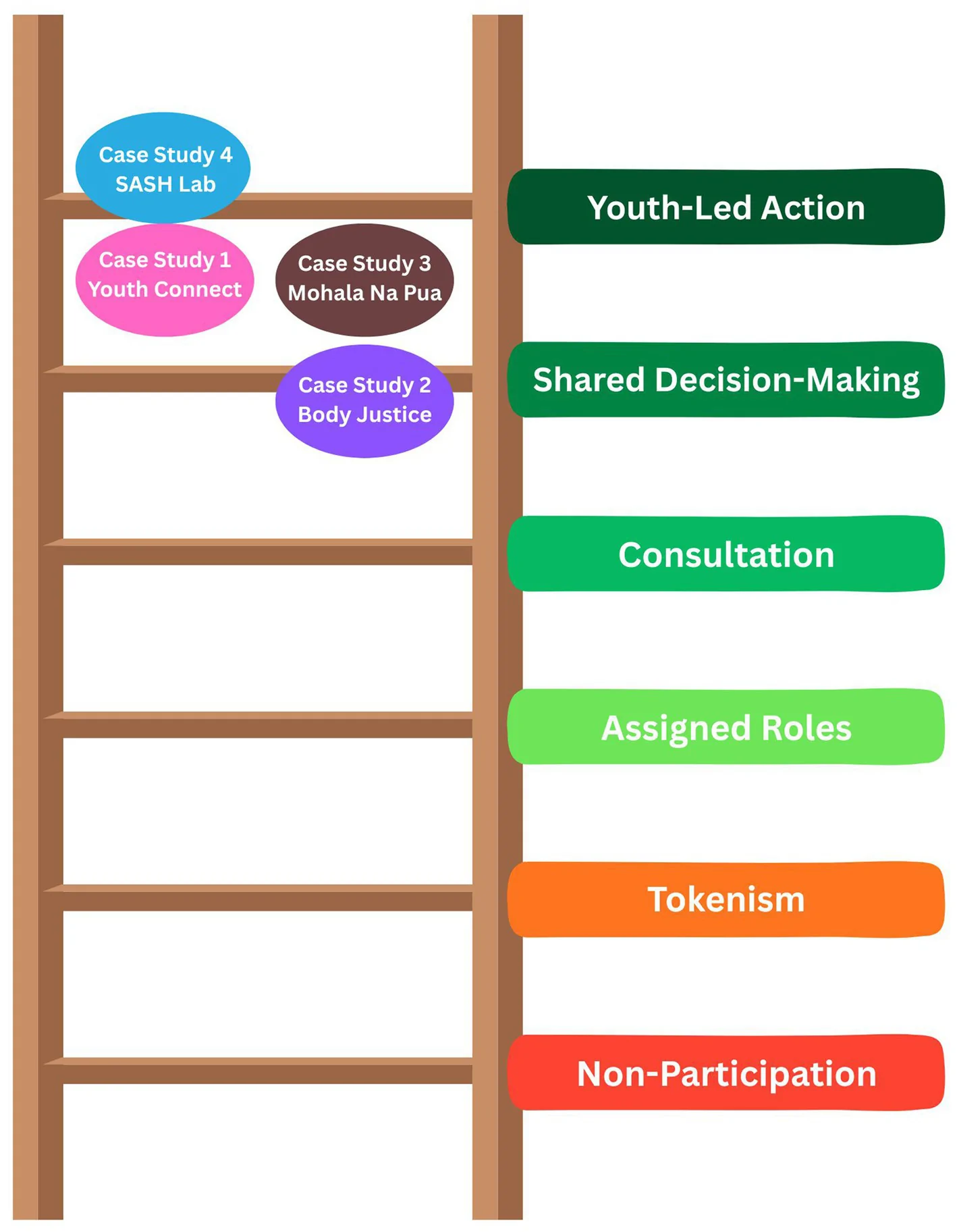
Community-based case studies on the ladders of participation.

This section summarizes four unique participatory projects representing diverse geographies and adolescent populations in the US. The case studies range from youth-adult partnered to youth-led, clustering at the top levels of participation in Hart’s ladder as represented in Figure 1 (16). The studies also demonstrate the flexible framework of participatory research that addresses a range of behavioral health disparities (Table 1).

**Table 1:** Youth-centered participatory project summaries.

Project name	State	Behavioral health disparity focus	Outcomes	Setting	Summary
Youth connections for wellbeing project	CA	Social Media Initiation and Wellbeing for Latine students	Research skills and STEM engagement; Social media & mental health literacy	Virtual	The aim of this project was to pilot a virtual, bottom-up approach to investigating unique mental wellbeing risks and strengths of social media initiation and active use among Latine adolescent communities. With an adapted and abbreviated YPAR curriculum, this project explored ways to center youth perspectives and empower them to be the experts of their own lived digital experiences. This project included pedagogy around research study design and practice, interviews, data interpretation, and science communication skills.
The body justice project	WA	Body Image Intervention to address race, gender, and sexual orientation diversity	Body appreciation, appearance pressure, general well-being, school climate, and disordered eating attitudes and behavior	Middle School	The overarching goal of this project was to collaboratively design and implement a body image curriculum that reflected the diverse perspectives and lived experiences of middle school students, particularly around issues of disordered eating and appearance pressure. Grounded in YPAR principles like power-sharing and centering youth voices, the project engaged students as co-creators of a culturally responsive, evidence-informed intervention aimed at promoting body autonomy and inclusivity within a diverse school community.
Mohala Na Pua project	HI	Youth Substance Use Prevention for Native Hawaiian and Pacific Islander populations in rural areas	30-day substance use, risk, and resistance skills	Middle School	The aim of this project is to support the student-led redesign and implementation of a culturally-grounded substance use prevention program within Windward Oʻahu middle schools. To do so, the Hoʻokahua Summer Program engaged approximately 30 middle school students through a YPAR framework. This approach positions young people as active agents in shaping prevention strategies that directly impact their lives. By training students in inquiry, design thinking, and community-engaged research through four one-hour sessions, the program aims to create more relevant and effective prevention approaches while developing students’ critical thinking and civic engagement skills.
The SASH Lab at Yale summer youth research program	NJ	Youth Substance Use Prevention for Black youth living in urban areas	Research skill building and action on substance use prevention	University	The aim of this project was to train youth as citizen scientists in New Jersey on public health methods and machine learning/AI to reduce youth substance use in urban communities. The curriculum centered on introduction or public health, community organizing, youth substance use, geospatial analysis, social media use as a prevention model, machine learning/AI and data analysis. All methods are centered around substance use in which youth learned over the course of 6 weeks how youth substance use is studied in research and how it can impact their communities.

### 2.1 Case #1: youth connections for wellbeing project (California)

#### 2.1.1 Population and behavioral context

Latine youth in the US experience persistent disparities in access and use of behavioral health resources, driven by shortages of culturally responsive providers, stigma, and institutional mistrust (4, 20, 21). At the same time, social media has become a primary space where Latine youth seek information and connection, making it a promising digital setting for delivering culturally relevant and low-barrier psychosocial resource (2, 4, 18). During the early phase of COVID-19 (Jan-May 2021), 6 Latine high school students (4 females, 2 males, ages range from 14–19) from an afterschool STEM Clubhouse, ListoAmerica Inc. in Southern California, along with their Director (GM; adult community partner) built a partnership to pilot a virtual YPAR project that explored Latine youth experiences of mental wellbeing while using social media (21). The project was brought together with adult researchers (i.e., 1 PhD [MI], 1 graduate student [JMH], and 1 undergraduate research assistant). This 15-session rapid-response curriculum was virtually adapted from existing YPAR curricula (7, 22) and parsed into 3 key modules (Table 2). The approach fostered trust-building with adult researchers, bidirectional knowledge sharing, and empowered youth to conduct qualitative interviews to answer research questions they developed. Students were compensated $25 for each YPAR session to mirror practices of compensating researchers for their expertise and contributions to research.

**Table 2:** Adapted YPAR curriculum for teen social media use and wellbeing.

Modules	Session themes
1. Establishing Teen-Adult Partnership; Topic Exploration	Session 1: Introductions and Relationships Building Session 2: Trust Building: Sharing Lived Experiences
2. Identifying the Issue & Research Methods	Session 3–4: Identifying Key Issues in Teen Social Media Use and Mental Wellbeing Support Session 5: Overview of Research Methods Session 6–8: Interview Protocol Development and Piloting Session 9–10: Conduct Interviews – independent field work
3. Research Interpretation & Application	Session 11–12: Interpretation of Data Collection Session 12–13: Application of expertise – Survey Redevelopment Session 14: Application of expertise - Social Media Mental Health Campaign Focus Group Session 15: Wrap Up and Feedback Discussion**

**Presentation to the Broader Adult Research Group.

#### 2.1.2 Methodology: qualitative evaluation of research findings by youth

During sessions 3–4 (Table 2), youth identified key issues of social media and wellbeing among Latine youth based on their lived experiences. This guided the development of an interview protocol (sessions 6–8) with the adult research team and included: (1) how you present yourself on social media matters and personas differ, (2) influencer posts on social media have different effects for people, and (3) awareness that many teens use technology as a place to hide from reality. Individual and familial experiences were discussed when identifying specific challenges and an additional component on *how teens learn how to use social media* was addressed. Many noted older siblings play a large role in social media adoption and parents’ fear of use of these tools. This amplified tensions of interpreting adaptive and maladaptive views around how to use social media. The youth conducted 21 interviews through snowball sampling of their peer networks and verbal informed consent was built into the interview protocol.

*Gender, Race, Social Comparison, and Body Image.* As central themes emerged from the interview process, youth identified distinct gender-based experiences in their peer interviews. Female participants frequently reported unrealistic body image expectations on social media. In contrast, males noted they were unaffected but often come across sexualized content more often than girls. Females agreed that influencer disingenuous messaging and negative comments can affect their wellbeing, while males were less affected. Both males and females endorsed passively and actively engaging in content that aligned with their interests as ways to support their wellbeing.

“A negative aspect of social media is comparing themselves with others and seeing this ideal body, and then they start doubting themselves…But in another interview that I did, they also said that one of the influencers they followed use that platform to bring awareness to those things saying it does not matter what you look like.” –Youth Researcher #3.

Youth also identified how sociocultural factors influence social media engagement and how it affected their views on their own cultures and society. For many youths of color, the pressure to conform on society standards often dictated what they felt comfortable posting. Despite these pressures, protective factors were also identified, such as following influencers who promote body positivity, which allows them to curate content that mirror their own lived experiences that fosters wellbeing. As one interviewee explained, viewing content that parallels their own experience allows control for what they experience and how they feel on social media.

“For like ethnic people, I think [what to post on social media] can also have to do a lot with trying to [fit] into America’s society or like that kind of standard. Like my friend was like mentioning that she did not want to wear that kind of dress, because she thought it’d be like weird to post about it.” –Youth Researcher #4.

#### 2.1.3 Outcomes and lessons learned

This pilot primarily advanced the individual-level processes of youth participatory approaches by positioning Latine adolescents as the experts of their own lived experiences and strengthening their social media and mental health literacy. Through serving as researchers by developing protocols, conducting interviews, and interpreting findings, youth built critical language and analysis skills to recognize how social media shaped the risks and opportunities it has on their wellbeing. This project also addressed the stigma and literacy-related dimensions of behavioral health disparities in a digital space that Latine youth frequent. Participants were then able to identify potential access points to culturally responsive mental health resources that would otherwise pose more challenges in the offline world (4, 21).

Youth then applied their expertise to existing tools. Drawing on the protective and risk patterns identified in their interviews, they provided feedback that refined the study’s survey design and informed a social media mental health campaign (Sessions 12–14). They concluded the pilot project by sharing critical insights from the findings and process in a community dialog with academic and community partners (Session 15), reflecting early advocacy in which youth voiced culturally grounded recommendations to the adults and systems engaging them. Limitations of the virtual-only setting included sustaining long-term engagement online and screen fatigue. However, given the nature of pandemic safety considerations, this project served as a proof of concept that virtual settings can build inclusive space for youth participation.

### 2.2 Case #2: the body justice project (Washington)

#### 2.2.1 Population and behavioral context

A team of middle school students, staff, and university-based researchers convened to engage in a youth-centered research project to co-design an evidence-informed body image intervention in September 2021. The collaboration was initiated by a local middle school who sought support around students experiencing disordered eating and body image concerns. The middle school serves around 600 students per year who are racially and economically diverse. Students often face systemic obstacles in accessing evidence-based interventions that addresses healthy body image, which are often not adapted to the unique needs of diverse school contexts (23). Interventions also often neglect to center the body image experiences of racially, sexually, and gender diverse young people who are at high risk for body image concerns, disordered eating, and eating disorders (24–27).

#### 2.2.2 Methodology: co-design process of body justice project

The participatory co-design process was intentionally cultivated within large and diverse advisory teams across the middle school and university sites in terms of gender identity, sexual orientation, and race. It included 7 staff members (3 paraeducators, 2 school counselors, and an assistant principal) and 17 middle school students (6th, 7th, and 8th graders), 3 undergraduate, and 2 faculty researchers within a psychology department. Separate adult and youth advisory teams met bi-weekly throughout the school year to design the body image curriculum and create a sustainable evaluation and implementation plan. Several of the university-based researchers and middle school staff members attended both the adult and youth advisory meetings, sharing information back and forth between the teams. Participatory principles—including power sharing, centering diverse youth voices, and challenging adult-youth hierarchies—shaped team meetings and the curriculum’s content and structure. The youth team shaped key activities, and staff co-wrote the program to reflect the advisory teams’ different perspectives. The final *Body Justice Project* curriculum was piloted with one 6th, two 7th, and one 8th grade class. Trained college students (1 per class) co-led the curriculum with youth leaders (1–2 per class) who participated in a youth leadership cohort. The intervention included eight 45-min lessons delivered within homeroom periods. The curriculum includes didactic (media literacy) and interactive components anchored in cognitive dissonance theory and covers four themes and learning goals (Table 3). Post-pilot revisions incorporated teacher, student, and program facilitators’ feedback (23) and created a sustainable implementation plan to deliver the *Body Justice* lessons with 7th graders each year. School staff would lead implementation efforts (coordination, communication with teachers and families) and the university would lead evaluation activities, assessing the impact on body image outcomes (e.g., body appreciation, appearance pressure), general well-being (e.g., self-worth), school climate (e.g., perceptions of appearance-based teasing), and disordered eating attitudes and behavior.

**Table 3:** Body justice project program framework.

Lesson theme	Core focus	Learning goals
Cultural appearance ideals	Critiquing narrow cultural norms of attractiveness	Recognize exclusionary standards; define health flexibly and individually
Diet culture & non-diet nutrition	Understanding size-inclusive health and intuitive eating	Deconstruct diet myths; explore how food reflects culture
Social media & appearance pressure	Analyzing the influence of media on body image	Critique advertising and redesign social feeds to promote body diversity
Body autonomy	Building body compassion and boundary-setting skills	Practice body appreciation and respect for diverse bodies

#### 2.2.3 Outcomes and lessons learned: evaluation and perspectives from community partners

This partnership is now in its fourth year of annual implementation and evaluation. The first 2 years used youth leader cohorts to co-facilitate lessons along with college leaders. Youth co-leadership paused the third year due to budget cuts and the loss of key advisory team members with close student relationships. Across the first 3 years, 333 7th grade students (*M* age = 12.44 years, *SD* = 0.51) have participated in *Body Justice* lessons across 20 classrooms (53% cisgender boys, 36% cisgender girls, 12% gender diverse students). Approximately half were students of color (49%). Twenty trained college leaders have co-led the curriculum (ages 19–25; 55% students of color; 70% cisgender women, 10% cisgender men, 20% gender diverse) with 31 youth leaders.

Many voices worked together to build and sustain this participatory project and school-based health promotion curriculum. Key strengths identified by researcher (ACC) and community member (CB) include the initial co-design team with more than two dozen members, which was also challenging at times (e.g., to coordinate schedules). Success was maintained by school staff who were both passionate about the topic of body image and well-connected and trusted by the students. Including diverse student voices in the co-design process allowed us to address the behavioral health disparities that exist within disordered eating and body image concerns, where marginalized young people are at higher risk than their peers with majority social identities. The students met regularly to share their worries and hopes for making the world a safer place to exist within diverse bodies, which was crucial to the process. Without staff and students’ enthusiasm, productive meetings would be difficult to sustain regularly and over time. The large team created a program that reflected important and varied perspectives. However, budget and staff cuts have changed the landscape of our collaboration over time, as non-teacher staff positions have been reduced. It may be hard to replicate a school-based participatory collaboration without staff who are willing to take significant ownership and encourage sustained student participation.

The ability to constantly revise and update the curriculum so the lessons stay current has also been key to our long-term success. Our reciprocal feedback process includes curriculum revisions based on facilitator reflection and staff and youth feedback each year. Data and impact are shared back with the school to provide *Body Justice Project*’s social–emotional success and to inform subsequent iterations. School administration favors the university collaboration as it allows students to receive a research-based curriculum without requiring extra time or effort from teachers. Our novel solution of using trained college facilitators (and youth co-facilitators when possible) allows teacher to focus on classroom management and helps to relieve burden on teacher time, which was a priority for the school from the start. In the fourth year, implementation time is relatively minimal and is a “well-oiled machine” from the school’s perspective (CB), with trust that college student facilitators will be well-trained and that the curriculum will reflect the latest evidence around body image health promotion.

### 2.3 Case #3: Mohala Na Pua project (Hawaiʻi)

#### 2.3.1 Population and behavioral context

The Mohala Na Pua project uses community-led implementation strategies to sustain a culturally grounded substance use prevention program, Ho‘ouna Pono (28). The program has been developed over the past 20 years with Hawai‘i Island intermediate school students to identify common drug offer scenarios that serve as the foundation for building resistance skills in youth. Native Hawaiian and Pacific Islander youth disproportionately suffer from higher rates of substance use, and this is more pronounced in rural areas (28). In 2022, Hawai‘i State Department of Education leadership reached out to the research team to spread Ho‘ouna Pono outside Hawai‘i Island to Oʻahu. Leadership was clear – a randomized controlled trial was not the solution to an already taxed school system and teachers (29). Instead, the research and education team collaborated to apply for a National Institute on Drug Abuse award that would put youth and communities in the lead, which was awarded in 2024. Through innovation tournaments (i.e., design sprints), youth would lead the redesign needed to meet Oʻahu students’ needs. The Hoʻokahua Summer Program was conducted in 2025 with middle school students in Windward Oʻahu to pilot the Ho‘ouna Pono redesign using youth co-design framework. The program was designed to position students as co-researchers and co-designers by providing students with the relevant skills and experience to redesign Hoʻouna Pono and create an implementation plan specific to their school.

#### 2.3.2 Methodology: Hoʻouna Pono program curriculum and reflections

Approximately 30 middle school students participated in four one-hour sessions that introduced them to the Hoʻouna Pono program, research process, design thinking, and storytelling (see Table 4). Each session was thoughtfully designed to reflect key youth co-design principles: (1) *inquiry*—train students to ask questions and gather evidence to understand the world around them, (2) *participation—*encourage students to generate innovative recommendations and solutions to the issues they face, and (3) *transformation*—use student recommendations to improve programs that impact their lives. Community partners were an integral part of designing the session objectives and materials. Early career research assistants lead session content delivery to students. Following each session, students, teachers, community partners, and Mohala Na Pua team members provided feedback to refine materials and processes.

**Table 4:** Mohala Na Pua - Hoʻokahua summer program session overview.

Session topic	Activities	Key takeaways
Mohala Na Pua/Hoʻouna Pono overview	*Pilina (connection) exercise:* Students discussed their position in their lineup of siblings and how this has impacted them. *Mohala Na Pua/Hoʻouna Pono slideshow presentation:* Slideshow presentation on Mohala Na Pua and Hoʻouna Pono. *Student partner interview:* Students interviewed each other on topics related to their name, memories, and feedback on a newly developed Hoʻouna Pono video.	1: Cooperative learning formats can be instrumental in sustaining student attention and active participation. 2: Tangible, low-cost incentives such as snacks can be effective in promoting student engagement. 3: Video content featuring relatable narratives and characters proves particularly engaging for students, indicating that visual storytelling may strengthen connection to prevention messaging.
Substance use/Hoʻouna Pono	*Hoʻouna Pono lesson:* Students participated in a Hoʻouna Pono lesson that depicted receiving an alcohol offer from a family member. *Lesson feedback:* Students identified what they learned and how they would adapt the lesson.	1: Providing feedback/coaching to other students enhances learning and group cohesion. *“I think he should tell the mom and give his little brother a talk about why he should not do that.”* 2: Teacher facilitation can be strengthened through the provision of supplementary slide deck materials that outline key components of the Hoʻouna Pono lessons.
Substance use/Hoʻouna Pono	*Hoʻouna Pono lesson:* Students participate in a Hoʻouna Pono lesson that depicts being asked to hide a friend’s e-cigarette device. *Lesson feedback:* Students identified what they learned from and what they would change about the lesson.	1: Gamified learning experiences, such as an online escape room activity, provides students with authentic opportunities to practice refusal skills and engage in reflective analysis of their decision-making processes. *“I was successful in saying no because I stood up for myself and I [brought] up how it would affect my sports and family.”* 2: Slideshow presentations combined with comprehensive instructional guidance enable facilitators to deliver lessons consistently and effectively.
Empathy Maps & Moʻolelo (Storytelling)	*Hoʻouna Pono BTS video:* Students watched a video showcasing the process behind creating the Hoʻouna Pono video. *Empathy maps:* Students complete an empathy map identifying what they might say, think, do, and feel if offered drugs. *Moʻolelo & reflections:* Students learned about Indigenous storytelling and reflected on what they learned throughout the Hoʻokahua program.	1: A holistic approach to refusal skill training (addressing thoughts, feelings, words, and actions) can be effective in helping students develop comprehensive response strategies to drug offer situations. Say: *“No thank you, I’m not into that stuff”* Think: *“Should I tell the teacher or not”* Do: *“Run away or tell an adult about it”* Feel: *“I would feel upset or scared”* 2: Tactile and interactive learning experiences prove particularly engaging for students, indicating that expanded use of hands-on activities could strengthen overall program appeal and participation.

#### 2.3.3 Outcomes and lessons learned

By positioning youth as active knowledge producers, the Hoʻokahua Summer Program advanced prevention in two ways: it strengthened students’ individual resistance and decision-making skills while simultaneously generating youth-driven insights to transform the Ho‘ouna Pono program, teaching strategies, and school-level implementation. Integrating storytelling also reinforced cultural identity as s developmental asset and addressing substance use disparities with the cultural strengths among Native Hawaiian youth. Limitations to this approach include timing and resources needed to use instructional time for research purposes. Originally, the team had proposed a six-session design sprint which was shortened to four following concerns from leadership that the length would detract from educational goals. To address this, more effort has been focused on understanding core educational goals (e.g., math, literacy) that can be highlighted in the design sprint approach. Despite these limitations, this project demonstrates how youth co-design can both empower young people and systematically improve prevention programming in culturally and contextually relevant ways.

### 2.4 Case #4: the SASH lab at Yale summer youth research program (New Jersey)

#### 2.4.1 Population and behavioral context

The Substances and Sexual Health (SASH) Lab at Yale University established the Summer Youth Research Program in New Jersey in 2024 in collaboration with the lab’s graduate students, postdoctoral and research fellows, and scientists. Urban youth in racial-ethnic minority communities encounter significant barriers to behavioral health resources and programming, such as cost, transportation, and psychoeducation access, which heightens risks for substance use and risky sexual behavior during adolescence (20). New Jersey was chosen as a site due to the lab principal investigator’s existing community-based participatory studies and community partnerships in the state. In July 2024, 23 New Jersey youth participated in a six-week immersive program rooted in YPAR principles and 24 youth were enrolled in 2025’s cohort. The program was designed by a research team with extensive experience conducting community-based youth substance use prevention studies in urban cities in New Jersey and informed by the principal investigator’s (IO) long-standing commitment to the state. Recognizing that many urban youths face barriers such as transportation, cost, or lack of access to information, that prevent participation in elite, traditional university-based summer programs, the SASH Lab created an accessible, community-centered alternative by bringing a summer program to the community. A distinctive feature of this model is its geographic reach. Although Yale University is based in New Haven, the SASH Lab team consistently travels to Paterson—approximately 90 miles away—to conduct programming, attend community events, and meet with families.

#### 2.4.2 Implementation: SASH lab training to action

The six-week program was centered on training the next generation in youth substance use prevention and also served as a form of capacity building. The curriculum centered on introductory lessons in research methods, public health, and core data science concepts, including applications of machine learning and artificial intelligence to substance use prevention and broader community health challenges. Yale faculty, graduate students, and research staff lead interactive workshops, while local NJ based university partners provide centrally located spaces to ensure accessibility. Youth participants worked in small teams daily to pursue community-driven research questions and receive close mentorship from graduate students and faculty. The program culminates in youth-led data projects that were presented to local stakeholders and city leaders, positioning teens as knowledge producers and advocates in their own communities. At the conclusion of the first cohort, all participants were invited to join the SASH Lab at Yale as teen citizen scientists (30). Our youth advisory board and coalition members review study materials for cultural relevance and provide strategic input on ongoing projects. Through these experiences, youth not only contribute to research translation but also become active agents of change, applying their scientific and advocacy training to improve health communication and strengthen protective community norms.

#### 2.4.3 Outcomes and lessons learned

Engaging youth as citizen scientists bridged the gap between urban youth and academic institutions which transformed participation into behavioral health promotion youth leadership that overcomes many systemic barriers to health engagement in urban settings. In addition, 14 of the graduates from the program have continued in this role and have formed a New Jersey youth substance use prevention coalition within the lab that advises ongoing research activities and advocates for substance use prevention in their own cities. In its second cohort, which began in June 2025 and accepted 24 teens, the program has successfully trained 47 teens representing over 10 cities in New Jersey. Early lessons underscore both the impact of youth being changemakers in their own communities and challenges of sustaining such an intensive program, particularly given the high costs of delivery and the limited funding opportunities available for racial-ethnic minority communities.

To further nurture youth leadership, the SASH Lab held regular monthly office hours with coalition members and summer program participants, which provided structured mentorship and technical assistance as they developed substance use and mental health campaigns for their own schools and neighborhoods. Empowering youth in this way has major advantages as they are able to reach their peers in ways that scientists are unable to. Many participants have gone on to launch school-based prevention clubs, deliver classroom presentations, and collaborate with local organizations to sustain their initiatives beyond the summer program.

## 3 Discussion and lessons learned

### 3.1 Strengths of present adolescent behavioral health case studies

There remains a significant gap in integrating youth-centered research that addresses persistent behavioral health disparities (11, 17). While traditional intervention work often focuses on clinical symptom reduction, the youth-centered approach presented across the four cases target proximal behavioral health outcomes related to Latine digital stress (Case #1), body image and resiliency among racial, gender, and sexual minorities (Case #2), substance use prevention through storytelling and redesign (Case #3), and substance use prevention with data science among urban youth (Case #4). Strengths lie in the contextual and cultural considerations that influenced the participatory trust and relationships as well as building sustainable research and programming. Namely, these four case studies highlight health disparities in diverse social dimensions such as race, economic diversity, and geographic region. These perspectives highlight critical considerations such as participatory research partnership building, sustainability, time, and team creation that promotes bidirectional knowledge transfer for shared decision-making between adults and youth when applying youth voice in various ways throughout the research process. It also serves as a foundation for future empirical and theoretical research to develop more comprehensive youth participatory frameworks for behavioral health disparities.

#### 3.1.1 Community-academic partnership

Community-academic partnerships are essential for bridging the gap between research and practice and accelerate evidence-based interventions into real-world settings (31, 32). Investing time early to build trust through clear communication is critical for community partners. Goals and value alignment are key to ensuring trust, along with building realistic timelines for long-term projects (31–33). Furthermore, community-academic partnerships ensure that youth not only inform research priorities through their lived experiences but also acquire concrete, transferrable skills in data collection, analysis, dissemination, and advocacy as evident in Cases #1 and #4. Youth analyzed data with researchers and interpreted findings to share at university sponsored conferences, community town halls, and infographics for use in schools and social media. In Case #2, training in leadership and public presentation skills led to student-initiated projects and a podcast.

Youth-centered research such as YPAR transforms the traditional research participation trajectory from passive involvement to active, skill-building engagement by situating youth as both co-researchers and emerging leaders in addressing behavioral health disparities (6, 20). This model produces dual benefits: researchers gain nuanced, context-specific insights into community and adolescent needs, while youth experience psychological empowerment, acquire practical competencies, and professional experiences that enhance their educational and career outcomes. These competencies translate into immediate empowerment that can be applied to professional development opportunities that strengthen youth academic and career trajectories. The opportunity for youth to work with university staff encourages the next generation of scholars toward higher education while maintaining a mutually beneficial relationship. Cheng and colleagues (34) note the importance of establishing professional development goals to highlight the importance of youth’s success, which truly fosters a bidirectional relationship.

Collectively, these cases illustrate how to meet youth and communities where they are geographically, institutionally, and relationally - in community hubs (Cases #1 and #4), virtual spaces (Case #1), or intermediate school campuses (Case #2 and #3). This engagement also reflects an intentional commitment to community-based research that challenges traditional academic hierarchies. Researchers being present and implementing shared work within the community signals respect, accountability, and authentic partnership, living out the value of community expertise. Youth and parents highlighted how meaningful it is to have researchers come to *their* neighborhoods, noting that this presence validates their experiences and builds trust in the research process.

#### 3.1.2 Contextual considerations and adaptability

The cases also revealed the importance of centering geography and culture for participants. Youth-centered approaches to solution building encounter natural boundaries in resources. For example, urban settings may have more access to technology and science expertise (Case #1) whereas rural areas may have more access to stronger knit communities and shared resources (Cases #2, 3, and 4). Taking these nuances into consideration allows participatory research to culturally adapt for norms and practices within communities as solutions are co-designed. To be explicit, this approach celebrates a collectivist orientation to wellness that is lacking in western psychological research.

Impacts of this work extends beyond data collection or intervention testing. Through youth participatory approaches, youth become credible messengers who disseminate prevention and health promotion messages in their own schools, peer networks, communities and families—reaching spaces that academic institutions could not access on their own (19). This multiplier effect not only enhances community-level outcomes but also contributes to more sustainable models of public health implementation. By investing time, travel, and institutional resources in maintaining these relationships, these cases demonstrated that community-engaged scholarship have promise to address behavioral health disparities in rural and urban contexts.

### 3.2 Limitations, challenges, and future considerations

Although these case studies demonstrate the strengths of centering youth voice in the process of addressing behavioral health disparities, this work is not without limitations. The contextualized nature of each project—while essential for meeting communities where they are—impacts generalizability and introduces methodological variability that can complicate cross-study comparisons. Furthermore, as these projects were often resource-intensive and focused on immediate community needs, the approach may be constrained by environmental structure (school vs. out-of-school), researcher capacity (sabbatical opportunity – Case #2), and can vary in short-term (Case Study #1, 3) versus longitudinal (Case Study #2, 4) behavioral shifts and academic opportunities. However, these limitations serve as a critical challenge to the field of behavioral health research to adopt more youth-led co-design research. A movement toward youth-centered methods provides opportunities to develop non-traditional research that values community relevance and youth agency to address health disparities.

#### 3.2.1 Resourcing, time, and access

A considerable amount of time (months to years) was invested in connecting with community partners, evaluating needs, and creating a study. Many youth-centered researchers argue that the time invested is necessary, although the amount of time devoted to developing and nurturing these partnerships can be challenging. To mitigate the barriers, teams uncertain about engaging in a years-long partnership can start small (e.g., developing a community-university advisory board to guide a project or policy, engaging in community listening sessions, engaging in program evaluation activities to pair community agency needs with researcher skillsets). Another common criticism is that the research project duration is only a portion of the time invested. For example, graduate students may not be able to execute long-term participatory projects given the restraints of their program and the necessity of moving to different phase of training. Direct communication about collaboration duration contributes to community-academic relationships.

Another pressing challenge is youth retention, interest, and engagement. It is not always a guarantee that compensation will be enough for them to engage in research. Creating multiple engagement points across modalities (e.g., in person, online) that align with youth motivations can help to sustain collective engagement over time. Furthermore, integrating youth-centered research activities within existing programs provided a natural audience and structure that helped facilitate participation, which highlights a strength to meet youth where they are.

#### 3.2.2 Adapting to sociopolitical climate

Youth engaged research orientations are often influenced by the sociopolitical climate and require ongoing commitment to cultural responsiveness, equity, and reflexivity. Funding limitations and institutional parameters often constrain projects that strive to maintain intentional community partnerships. These challenges are paralleled with persistent inequities to mental health care, further justifying the need for culturally grounded and community-driven research practices. Despite these challenges, organizations such as the National Academies of Sciences, Engineering, and Medicine remain champions of YPAR to identify priorities for youth wellbeing (35).

Moreover, obstacles around community and youth-centered applied research are situated in the broader historical barriers within psychological research which still underrepresent marginalized communities. Youth-centered methods extend ongoing efforts to decolonize research through community-based participatory research frameworks and liberation psychology (12). Adolescents as co-researchers and advocates whose lived experience reveal the potential for long-term societal changes (e.g., shifting attitudes toward immigrant communities) shape collective and intergenerational wellbeing (14). Academic publication and paid firewalls limit research access to community spaces. Opportunities to disseminate community impact findings (e.g., number of people reached) in non-academic publications may be a method to increase access.

#### 3.2.3 Participatory research ethics and evaluation

Ethical considerations pose unique challenges for participatory research with youth. Although the IRB plays a critical role in protecting participants, the current structures often constrain the flexibility that community-engaged research requires. Case #2 took an approach which avoided an ethics or institutional review board (IRB) application for its co-design phase and only collected internal feedback to guide future programming. This enabled youth and school partners to shape the process organically without being bound to predetermined methods – a decision made possible due to the lead investigator’s (ACC) ability to forgo a year of academic publishing, a privilege not available to all academics. Case #1 leveraged opportunities with IRB, such as verbal, multilingual phone consent with families whose concerns also centered around trust with institutions in multi-status homes. While the adaptation in this IRB application limited the dissemination options of the teen researchers’ findings, safety and trust were upheld.

These experiences highlight that ethical rigor in youth-adult partnered research may not be able to solely rely on institutional compliance, but also must include transparency with partners, equitable compensation, and shared decision making about data ownership, program evaluation, and dissemination practices (18). Expanding IRB standards to acknowledge co-design, flexible consenting process, and consideration of community contexts could improve the inclusion and equity for an increasingly diverse youth population. Reframing ethics as an iterative and collaborative process may strengthen both community trust and scientific integrity.

## 4 Conclusion

The goal of this paper is to serve as a guide for research and community teams who wish to co-create sustainable programs to reduce health disparities and wellbeing alongside young people. There remains a gap in behavioral health disparities work that adapts youth-centered participatory approaches for research and intervention implementation (11, 12). Authored by both researchers and community partners, these case studies provide actionable frameworks for teams that can be scaled and modified according to context, implementation team, and cultural and mental health considerations. We highlight flexible shared power and decision making frameworks that elevated youth voices to understand and build behavioral health interventions guided by the Hart’s Ladders of Youth Participation (16). These exemplars demonstrate feasibility and impact of partnerships grounded in trust and shared power.

Sustaining such partnerships requires time, financial investment, and procedural flexibility that values relational accountability and cultural humility. A call to address the youth mental health crisis in the US (1) must center diverse youth voices in behavioral health research to prevent additional mental health risks (4, 8, 19, 20). Embedding YPAR and youth-centered practices into psychology and public health fields can bridge how communities, researchers, and institutions collaborate, shifting from doing research *for* youth to evidence-based solution building *with* youth for healthier future generations.

## Data Availability

The raw data supporting the conclusions of this article will be made available by the authors, without undue reservation.

## References

[1] The U.S. Surgeon General’s Advisory Protecting Youth Mental Health (2022). Available online at: https://www.hhs.gov/sites/default/files/surgeon-general-youth-mental-health-advisory.pdf (Accessed October 10, 2025).

[2] Hill EK Bond L Weisz JR Patel V. Addressing the adolescent mental health-care gap in the United States. J Adolesc Health. (2025) 77:1014–6. doi: 10.1016/j.jadohealth.2025.08.007, 41003442

[3] Sawyer S Azzopardi P Wickremarathne D Patton G. The age of adolescence. Lancet Child Adolesc Health. (2018) 2:223–8. doi: 10.1016/S2352-4642(18)30022-1, 30169257

[4] Rodgers CRR Flores MW Bassey O Augenblick JM Cook BL. Racial/ethnic disparity trends in children’s mental health care access and expenditures from 2010-2017: disparities remain despite sweeping policy reform. J Am Acad Child Adolesc Psychiatry. (2022) 61:915–25. doi: 10.1016/j.jaac.2021.09.420, 34627995 PMC8986880

[5] Farmer TW Serpell Z Scott LA DeVlieger SE Brooks DS Hamm JV. The developmental dynamics of emotional and behavioral difficulties of youth of color: systemic oppression, correlated constraints, and the need for targeted universalism. J Emot Behav Disord. (2022) 30:71–85. doi: 10.1177/10634266211068892

[6] Ozer EJ. Youth-led participatory action research: overview and potential for enhancing adolescent development. Child Dev Perspect. (2017) 11:173–7. doi: 10.1111/cdep.12228

[7] Ozer EJ Abraczinskas M Duarte C Mathur R Ballard PJ Gibbs L . Youth participatory approaches and health equity: conceptualization and integrative review. Am J Community Psychol. (2020) 66:267–78. doi: 10.1002/ajcp.12451, 32969506

[8] Rodríguez LF Brown TM. From voice to agency: guiding principles for participatory action research with youth. New Dir Youth Dev. (2009) 2009:19–34. doi: 10.1002/yd.312 19830799

[9] Newman BM Newman PR. "Summary". In: Newman BM Newman PR, editors. Theories of Adolescent Development. London: Academic Press (2020). p. 395–409. Available online at: https://www.sciencedirect.com/science/chapter/monograph/pii/B9780128154502099976

[10] Ross KM Tolan PH. "Positive youth development (PYD) in the digital age: expanding PYD to include digital settings". In: Dimitrova R Wiium N, editors. Handbook of Positive Youth Development: Advancing Research, Policy, and Practice in Global Contexts, Springer Series on Child and Family Studies. Cham, Switzerland: Springer International Publishing (2021). p. 531–48. doi: 10.1007/978-3-030-70262-5_35

[11] Anyon Y Bender K Kennedy H Dechants J. A systematic review of youth participatory action research (YPAR) in the United States: methodologies, youth outcomes, and future directions. Health Educ Behav. (2018) 45:865–78. doi: 10.1177/1090198118769357, 29749267

[12] Malott KM Smith-Yliniemi J Ali L Darazsdi Z. Decolonizing adolescent interventions: from marginalization to liberation. J Child Adolesc Couns. (2025) 11:175–98. doi: 10.1080/23727810.2025.2517521, 37339054

[13] Ryan RM Deci EL. Self-determination theory and the facilitation of intrinsic motivation, social development, and well-being. Am Psychol. (2000) 55:68–78. 11392867 10.1037//0003-066x.55.1.68

[14] Cahill H Dadvand B. Re-conceptualising youth participation: a framework to inform action. Child Youth Serv Rev. (2018) 95:243–53. doi: 10.1016/j.childyouth.2018.11.001

[15] Wong NT Zimmerman MA Parker EA. A typology of youth participation and empowerment for child and adolescent health promotion. Am J Community Psychol. (2010) 46:100–14. doi: 10.1007/s10464-010-9330-0, 20549334

[16] Hart RA. Children’s Participation: From tokenism to citizenship (1992) Available online at: https://www.unicef-irc.org/publications/100-childrens-participation-from-tokenism-to-citizenship.html (Accessed September 25, 2025).

[17] Stiles-Shields C Cummings C Montague E Plevinsky JM Psihogios AM Williams KDA. A call to action: using and extending human-centered design methodologies to improve mental and behavioral health equity. Front Digital Health. (2022) 4. doi: 10.3389/fdgth.2022.848052, 35547091 PMC9081673

[18] Hite B Berry T Theodorou E Sloper M. Evaluating youth programs with intention: integrating developmental research for a youth-centered approach. J Youth Dev. (2026) 21. doi: 10.5195/jyd.21.01.02

[19] Ozer EJ Sprague Martinez L Abraczinskas M Villa B Prata N. Toward integration of life course intervention and youth participatory action research. Pediatrics. (2022) 149:e2021053509H. doi: 10.1542/peds.2021-053509H, 35503322 PMC9847417

[20] Smith KE Acevedo-Duran R Lovell JL Castillo AV Pacheco VC. Youth are the experts! Youth participatory action research to address the adolescent mental health crisis. Health. (2024) 12:592. doi: 10.3390/healthcare12050592, 38470702 PMC10930985

[21] Hernandez JM Cabrera JD Ito M. "Youth participatory action research in Latinx adolescent social media use and wellbeing". In: TMS Proceedings 2021. Washington (DC): American Psychological Association (2021) p. 2.

[22] The Institute for Community Research’s Youth Partcipatory Action Research Curriculum. (2014). Available online at: https://www.oregon.gov/oha/PH/HEALTHYPEOPLEFAMILIES/YOUTH/Documents/CurriculumYPAR2014.pdf (Accessed October 27, 2025).

[23] Pascual S Martini A Gambito J Gemar C Bell E Delucio K . Developing a justice-focused body image program for U.S. middle schoolers: a school-based community-engaged research process. Eat Disord. (2024) 32:623–43. doi: 10.1080/10640266.2024.2328402, 38483795

[24] Howe CP Baik SY D’Adamo L Kouveliotes M Pan Z Monocello L . Examining prevalence and presentations of eating disorders across racial/ethnic groups in a national, population-based sample of college students. Int J Eat Disord. (2025) 58:1165–77. doi: 10.1002/eat.24427 40152153 PMC12140885

[25] Vázquez MM Fowler LA Zhu Y Grammer AC Fitzsimmons-Craft EE Lipson SK . Disparities in mental health symptoms among sexual and gender diverse subgroups in a national sample of college students. Psychol Sex Orientat Gend Divers. (2024). doi: 10.1037/sgd0000714, 40693689 PMC12221272

[26] Cheng ZH Perko VL Fuller- Marashi L Gau JM Stice E. Ethnic differences in eating disorder prevalence, risk factors, and predictive effects of risk factors among young women. Eat Behav. (2019) 32:23–30. doi: 10.1016/j.eatbeh.2018.11.004, 30529736 PMC6382562

[27] Nagata JM Stuart E Hur JO Panchal S Low P Chaphekar AV . Eating disorders in sexual and gender minority adolescents. Curr Psychiatry Rep. (2024) 26:340–50. doi: 10.1007/s11920-024-01508-1, 38829456 PMC11211184

[28] Okamoto SK Kulis SS Helm S Chin SK Hata J Hata E . An efficacy trial of the ho‘ouna Pono drug prevention curriculum: An evaluation of a culturally grounded substance abuse prevention program in rural Hawai‘i. Asian Am J Psychol. (2019) 10:239–48. doi: 10.1037/aap0000164, 32395199 PMC7213509

[29] Okamura KH Palafu T An K Marshall SM Chin SK Stern KA . “Allowing space for voice…all our voices”: understanding ho‘ouna Pono implementation through educational leadership perspectives in rural Hawai‘i schools. Sch Ment Heal. (2024) 16:793–807. doi: 10.1007/s12310-024-09660-y, 39464697 PMC11507294

[30] Asabor EN Aneni K Weerakoon S Opara I. Applying a community-engaged participatory machine learning model. Am J Community Psychol. (2024) 74:262–8. doi: 10.1002/ajcp.12765, 39279135 PMC12901535

[31] Israel BA Schulz AJ Parker EA Becker AB. Review of community-based research: assessing partnership approaches to improve public health. Annu Rev Public Health. (1998) 19:173–202. 9611617 10.1146/annurev.publhealth.19.1.173

[32] Wallerstein N Duran B. Community-based participatory research contributions to intervention research: the intersection of science and practice to improve health equity. Am J Public Health. (2010) 100:S40–6. doi: 10.2105/AJPH.2009.184036, 20147663 PMC2837458

[33] Khatib M Shah R Mendhe S Whisner CM Buman MP. Bridging the divide: barriers and facilitators to equitable community-academic partnerships in health research. Front Public Health. (2025) 13:1617908. doi: 10.3389/fpubh.2025.1617908, 41036127 PMC12479405

[34] Cheng M Levinson E Pierre J Reddy D Sattler K Siva O . Youth voices are needed now more than ever to improve adolescent sexual health research: lessons from five years with a youth advisory board. Sex Res Soc Policy. (2025). doi: 10.1007/s13178-025-01230-4, 41541233 PMC12803725

[35] National Academies (2025) Digital Tools for Youth Mental Health Summit. Available online at: https://www.nationalacademies.org/event/45370_11-2025_digital-tools-for-youth-mental-health-summit (Accessed October 31, 2025).

